# Scalable downstream method for the cyclic lipopetide jagaricin

**DOI:** 10.1002/elsc.202100079

**Published:** 2021-10-27

**Authors:** Jakob Stein, Nicolas Schlosser, Bettina Bardl, Gundela Peschel, Florian Meyer, Florian Kloss, Miriam A. Rosenbaum, Lars Regestein

**Affiliations:** ^1^ Bio Pilot Plant Leibniz Institute for Natural Product Research and Infection Biology Hans‐Knöll‐Institute Jena Germany; ^2^ Faculty of Biological Sciences Friedrich Schiller University Jena Jena Germany; ^3^ Transfer Group Anti‐Infectives Leibniz Institute for Natural Product Research and Infection Biology Hans‐Knöll‐Institute Jena Germany

**Keywords:** antifungal, antimicrobial, biosurfactant, *Janthinobacterium agaricidamnosum*, lipopeptides

## Abstract

Cyclic lipopeptides are substances with a high potential to act as antimicrobial agents. Jagaricin, produced by *Janthinobacterium agaricidamnosum* DSM 9628 and discovered in 2012, is a new member of this class with promising antifungal properties. However, further experiments to investigate future applications and/or conduct chemical derivatization to change properties and toxicity are impossible due to the limited access to jagaricin. Besides a high jagaricin concentration at the end of the fermentation process, a suitable downstream process is essential to generate appropriate amounts with the desired purity. In contrast to other amphiphilic molecules, jagaricin cannot be separated by foam fractionation since it is mainly attached to the surface of the microbial biomass. This technical report presents an overall process chain consisting of 11 individual steps to generate jagaricin in gram scale with a purity of over 95%.

## 1 INTRODUCTION

Lipopeptides belong to the diverse family of biosurfactants which are microbial‐derived surface‐active compounds. They are produced by various microorganisms such as *Pseudomonas*, *Bacillus*, and *Streptomyces* species [1, 2, 3]. Usually, lipopeptides consist of a hydrophilic peptide moiety attached to a hydrophobic tail residue resulting in an amphiphilic character [1, 4]. Owing to this characteristic, lipopeptides have various physicochemical properties such as emulsification/de‐emulsification, dispersing, foaming, viscosity reducing, solubilizing and mobilizing agents, and pore‐forming capacity [1]. Additionally, lipopeptides show diverse biological activities such as antimicrobial, antiviral, hemolytic, and insecticidal effects [1, 5, 6]. With properties such as eco‐friendliness, specificity, low toxicity, stability in varying environmental conditions, and chemical diversity, some microbial biosurfactants stand the chance of replacing synthetic surfactants in industrial applications soon [7].

**PRACTICAL APPLICATION**

The class of cyclic lipopeptides is of high interest due to their potential as antimicrobial agents. A broad spectrum of different cyclic lipopeptides has been investigated so far, with a strong focus on biosynthetic formation. This technical report shows the results of a scalable overall downstream process chain from crude fermentation broth to the pure product jagaricin. Based on this process, biomass‐attached cyclic lipopeptides can be produced in gram scale with purities higher than 95%.

An impressive subgroup of this class comprises cyclic lipopeptides with a considerable potential to serve as antimicrobial agents. One commercially available representative of this subgroup is the antibiotic daptomycin. It was isolated from *Streptomyces roseosporus* [8, 9] and shows rapid bactericidal killing of Gram‐positive pathogens. In 2012, Graupner et al. [10] identified the novel cyclic lipopeptide jagaricin (Figure S1) generated by *Janthinobacterium agaricidamnosum* DSM 9628. This substance is a main pathogenicity factor of the soft rot disease in the fungus *Agaricus bisporus* [11]. Moreover, first studies have shown that jagaricin is highly active against human pathogenic fungi such as *Candida albicans*, *Aspergillus fumigates*, and *Aspergillus terreus* [10]. Furthermore, it was proven that jagaricin has no or low plant toxicity at concentrations where the growth of prevalent phytopathogenic fungi is inhibited [12].

However, for all further research and application of jagaricin in a medical, pharmaceutical, agricultural or environmental context, appropriate amounts in gram scale are necessary with sufficient purity. To produce jagaricin, Schlosser et al. optimized and scaled up a bioprocess based on *J. agaricidamnosum* [13]. The fermentation broth of the scaled up bioprocess contains complex medium compounds and antifoam agent, which may challenge the development of a downstream process. Standard techniques for extraction from the supernatant are precipitation with acid or ammonium sulfate, solvent extraction, and foam fractionation [1, 4, 14]. Often the acid precipitation is followed by solvent extraction [1]. Ammonium sulfate precipitation is not as common as acid precipitation because the following dialysis step is needed to remove the salts [1, 15]. To achieve a higher purification grade, techniques such as ultrafiltration, ionic exchange chromatography, and adsorption‐desorption on resins or other matrices are used [1, 4]. Different chromatographic methods like high performance liquid chromatography (HPLC), hydrophobic interaction chromatography, and gel filtration are suitable to fractionate and purify lipopeptide compounds [1, 4]. A detailed overview of already published downstream processes of lipopeptides is given in Table 1.

**TABLE 1:** Overview of published downstream processes for the purification of lipopeptides

Lipopeptide	Purification steps	References
Fengycin‐like lipopeptide	1. Separation of the supernatant via centrifugation 2. Acid precipitation from supernatant 3. RP‐HPLC	[16]
Pseudofactin	1. Foam collection and centrifugation 2. Cell pellet was extracted with acetonitrile 3. Supernatant was boiled (precipitation) 4. Precipitate was extracted with organic solvents	[17]
Surfactin	1. Separation of the supernatant via centrifugation 2. Acid precipitation from supernatant 3. Solvent extraction from precipitate 4. Adsorption on HP‐20 resin	[15]
	1. Foam fractionation 2. Separation of the biomass from the foam via centrifugation 3. Ultrafiltration of cell‐free foam fractions	[18]
	1. Separation of the supernatant via centrifugation 2. Adding of ethanol and two‐stage ultrafiltration of the supernatant 3. Acid precipitation 4. Precipitate dissolved in NaOH solution 5. Adsorption/ion‐exchange with resins	[19]
C_14 _surfactin and C_16_ iturin like compounds	1. Separation of the supernatant via centrifugation 2a. Solvent extraction and thin‐layer chromatography (TLC) 2b. Acid precipitation and solid‐phase‐extraction 2c. Ammonium sulfate precipitation and gel filtration chromatography 3. RP‐HPLC	[20]
Unknown	1. Separation of the supernatant 2. Adsorption on resin (XAD‐4) 3. Chromatography steps (silica gel column chromatography, size exclusion chromatography, semi‐preparative RP‐HPLC, RP‐HPLC)	[21]
Viennamycin	1. Culture broth acidified to pH 4 2. Extraction with n‐butanol 3. Flash chromatography on RP18 column 4. Size exclusion chromatography	[22]

Previous studies have proven that jagaricin is secreted in the supernatant; however, larger amounts seem to be attached to the cell's surface. Especially this physicochemical property of the jagaricin molecule plays an important role in choosing the appropriate purification techniques [1, 23]. Therefore, already published downstream processes, which are designed to extract lipopeptides from the supernatant, are not directly applicable and need to be modified for jagaricin purification.

## 2 MATERIALS AND METHODS

### 2.1 Cultivation of *J. agaricidamnosum*

For the production of jagaricin, the wild‐type strain of *J. agaricidamnosum* DSM 9628 (DSMZ, Braunschweig, Germany) was used. The main culture process data are presented in Figure S2, and further details are published in Schlosser et al. [13].

### 2.2 Process units for jagaricin purification

**FIGURE 1:**
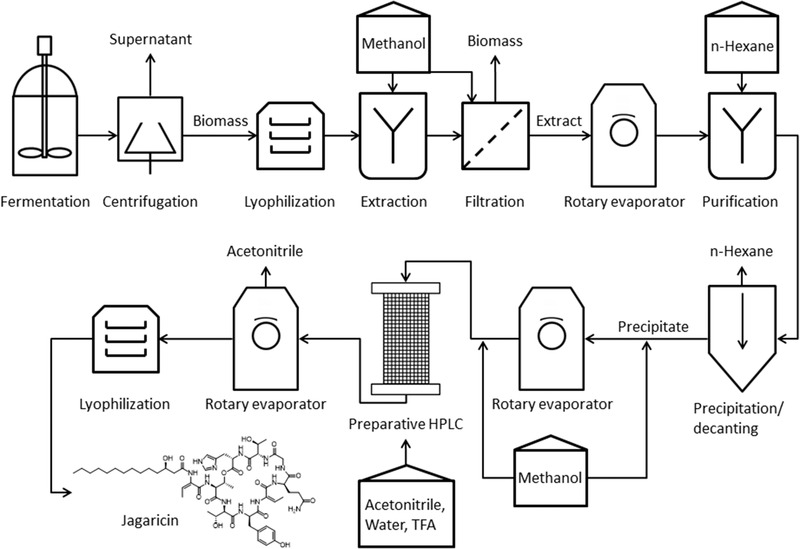
Flow chart for purification of the cyclic lipopeptide jagaricin from crude fermentation broth to the pure product

The following section contains only devices and chemicals named in order of the process chain (Figure 1). Further details of the individual process steps are described in Section 3. (1) After fermentation, the biomass was separated via centrifugation with 14,040 x *g* for 20 min (Avanti JXN‐26, Beckman Coulter, USA). The biomass was (2) lyophilized at ‐40°C for 60 h (Delta2‐24 LSC, Martin Christ Gefriertrocknungsanlagen GmbH, Germany). (3) For the extraction of jagaricin from the lyophilized biomass, methanol was used, and different solvent ratios were tested (10, 15, 20, and 40 mL g^–1^_biomass_). After extraction, the biomass was removed via vacuum filtration (Whatman cellulose filter, Merck, Germany). The jagaricin containing methanol phase was transferred to a rotary evaporator (Hei‐VAP Advantage, Heidolph, Germany) for volume reduction. (5) After reduction of the methanol phase, a defatting step had to be performed using n‐hexane as well as the following devices: Ultrasonic bath (Sonorex Super RK 100 H, Bandelin electronic GmbH & Co. KG, Germany), separator funnel (500 mL, Lenz Laborglas GmbH & Co. KG, Germany), Buchner funnel (Duran Buchner funnel, Merck, Germany) with cellulose filter (Whatman cellulose filter, Merck, Germany) and a filter funnel (POR 5, DWK Life Sciences GmbH, Germany). (6) The preparative HPLC was performed with a JASCO system. For separation, a C18 column was used (Eurospher EI 100–10 C18 Column 250 × 32 mm, Knauer Wissenschaftliche Geräte GmbH, Germany). The system was operated with 0.1% formic acid (FA) or 0.05% trifluoroacetic acid (TFA) in the solvents. The water phase was mixed with an increasing gradient of acetonitrile (Figure S4A). The flow rate was adjusted to 20 mL min^‐1^. Jagaricin was monitored at 254 nm (DAD‐Detector, MD‐2015 Plus, Jasco, Japan). All fractions were collected (SF‐3120, AdvanTec, Germany).

### 2.3 Analytical methods

For jagaricin quantification and determination of the purity, analytical UHPLC (Vanquish Horizon, Thermo Fisher Scientific, USA) equipped with a diode array detector and a Kinetex XB‐C18 column (Phenomenex, USA) was used. The solvent composition was changed over time containing water and acetonitrile with 0.1% trifluoroacetic acid. The gradient started at 10% of acetonitrile for 1 min, followed by a linear increase up to 98% within 4 min, and held for 3 min. The flow rate was adjusted to 0.4 ml min^–1^. Jagaricin was monitored at 222 nm.

To determine the correct weight of jagaricin, the electrostatic charge was neutralized with an ionization fan (Stat‐Fan IB‐8, Sartorius, Germany). Further investigations towards jagaricin purity were performed using nuclear magnetic resonance (NMR) spectroscopy (Bruker Avance III 600 MHz Spectrometer).

## 3 RESULTS AND DISCUSSION

### 3.1 Process chain

The presented process was developed according to the following restrictions: (1) The purity of the final purified jagaricin has to be higher than 95%, and (2) all process steps have to be scalable.

#### 3.1.1 Localization of jagaricin

Cyclic lipopeptides can be localized intracellular, extracellular in the supernatant, and/or attached to the cell wall. The analysis of the jagaricin location has shown that the extracellular content can be neglected since 15% are in the supernatant and 85% presumably bound at the cell surface. This result was expected due to the high hydrophobicity of the jagaricin structure (see Figure S1). The presented process chain is focused on the handling of the biomass–attached jagaricin. After removal of the supernatant, all biomass was lyophilized for 60 h at ‐40°C.

#### 3.1.2 Extraction and filtration

To characterize and optimize the extraction step, the volumetric methanol‐to‐biomass ratio and extraction time were investigated. The optimal methanol‐to‐biomass ratio is the minimal volume of methanol required for the total extraction of jagaricin. Four ratios (10, 15, 20, and 40 ml g^–1^_biomass_) were characterized. As visible in Figure S3A, 15 ml g^–1^_biomass_ is a suitable ratio for this extraction. The extraction kinetics presented in Figure S3B, clearly show the saturation of the methanol phase after 15 min. For separation of the jagaricin containing methanol phase from the microbial biomass, a dead‐end filtration was applied. Since moist remains in the filter cake, the losses of jagaricin were quantified with a relative value of 13%. Therefore, an additional flushing step with 5 ml g^–1^_biomass_ of methanol was performed to reduce the jagaricin losses to 3.6%. The filtrated extract was transferred to a rotary flask and concentrated till dryness. The final extract was stored at ‐20°C.

#### 3.1.3 Purification of extract (defatting step)

The crude jagaricin extract is associated with impurities from the initial fermentation broth, particularly antifoam agents and fatty acids. Thus, a defatting step with n‐hexane was carried out to remove lipophilic substances. Previous experiments (data not shown) had shown that jagaricin precipitates in ethyl acetate. Since n‐hexane is less polar, a similar behavior was assumed, and jagaricin was precipitated accordingly. The dried material was suspended in n‐hexane using an ultrasonic bath (5 min, 20‐22°C). After 2 h, precipitates were formed, which contained non‐soluble components like the aimed product jagaricin. Due to safety issues (explosion), the precipitates cannot be separated from n‐hexane with standard centrifuges. Thus, the clear supernatant was removed via decanting or filtration using a cellulose filter (11 μm pore size) or a glass frit (1.6 μm pore size). However, all three methods resulted in high losses of jagaricin between 32% and 43%.

#### 3.1.4 Preparative HPLC and lyophilization

The dried defatted precipitates were resolved in methanol before the final purification step was performed using a preparative HPLC. Investigated parameters comprised the loading capacity of the column and the type of acid used in the mobile phase. As published by Graupner et al. and Fischer et al., 0.1% of formic acid seems to be appropriate [10, 12]. However, obvious from Figure S4B, the utilization of formic acid resulted in a broad peak for jagaricin between 20 and 30 min. To increase the chromatographic resolution, formic acid was replaced by 0.05% trifluoroacetic acid, which decreases the pH due to a lower pKa value of 0.23. As visible in Figure S4B, jagaricin was eluted in a narrow window between 21 and 24 min, which significantly improved the previously published methods. The eluted product was separated into eight fractions (=30 s interval), which were transferred to rotary flasks for acetonitrile evaporation (70 mbar at 40°C). The final product was bottled and stored at room temperature (Figure S5).

### 3.2 Losses, purity, and optimization

**FIGURE 2:**
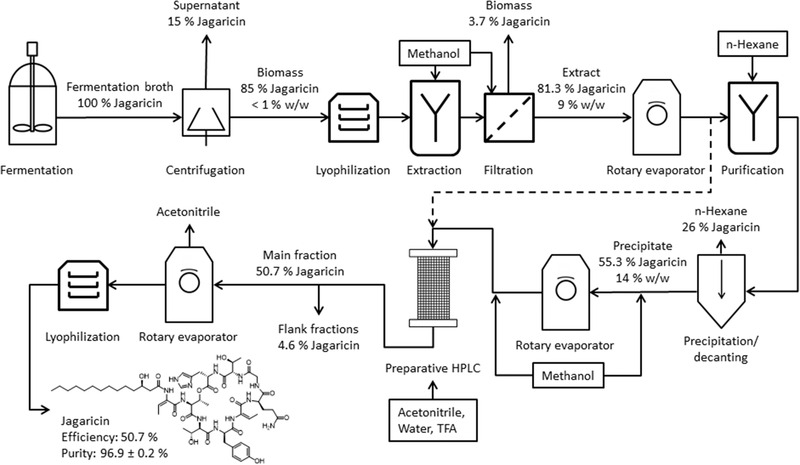
Losses of jagaricin related to the initial content after fermentation and purity of the jagaricin containing material. The dashed line represents an alternative theoretical process chain

To illustrate the losses of jagaricin and the increase of purity during the process, the scheme in Figure 1 was modified as depicted in Figure 2. The current loss of 15% of jagaricin through the supernatant could theoretically be avoided by extracting the product using ethyl acetate. However, large amounts of solvent would be necessary, which would also have to be processed in subsequent process steps. In addition, the supernatant contains all remaining compounds from the complex medium, which were not taken up by *J. agaricidamnosum* and would therefore give rise to further impurities in the final product. Consequently, processing the supernatant should have a very low priority in case of process optimization.

Highest potential for optimization is to be seen in the defatting step if the usage of an antifoam agent can be avoided in the fermentation process. Depending on foam formation and volume, a low aeration rate combined with a high stirring rate can reduce the foam formation rate. Moreover, aeration by increasing the headspace pressure and applying a membrane module for bubble‐free aeration can drastically reduce foam formation [24, 25]. Even in the case of antifoam usage, a shortening of the process chain, as indicated by the dashed line in Figure 2, should be evaluated. With the minor increase in purity from 9% to 14% in relation to the loss of 26% of jagaricin, all n‐hexane related steps are the most critical bottleneck in the overall process.

## 4 CONCLUDING REMARKS

This technical report presents a downstream process chain to purify the antifungal cyclic lipopeptide jagaricin from fermentation broth. Since jagaricin is mainly attached to the surface of the bacterial biomass, more process steps are necessary to achieve a purity over 95% compared to other published lipopeptide forming bioprocesses. Potential for optimization was identified in the defatting step to remove the antifoam agent by an n‐hexane extraction. Since there are alternative methods besides antifoam agents to avoid foam formation, there is a good chance to shorten the presented process chain and increase overall efficiency even more. Currently, a purity of 96.9% jagaricin and total efficiency of 50.7% can be achieved upon the process as presented.

## CONFLICT OF INTEREST

The authors declare no conflict of interest.

## Supporting information

Supporting information.

## Data Availability

The data that support the findings of this study are available from the corresponding author upon reasonable request.
