# Supplementary material for: Scalable downstream method for the cyclic lipopetide jagaricin
Source: Eng Life Sci. 2021 Oct 27;22(12):811–7. doi: 10.1002/elsc.202100079 (PMC9731587; doi:10.1002/elsc.202100079)
Supplement: Supplementary file 1 — Supporting information. [file ELSC-22-811-s001.pdf]

Supplementary Figures

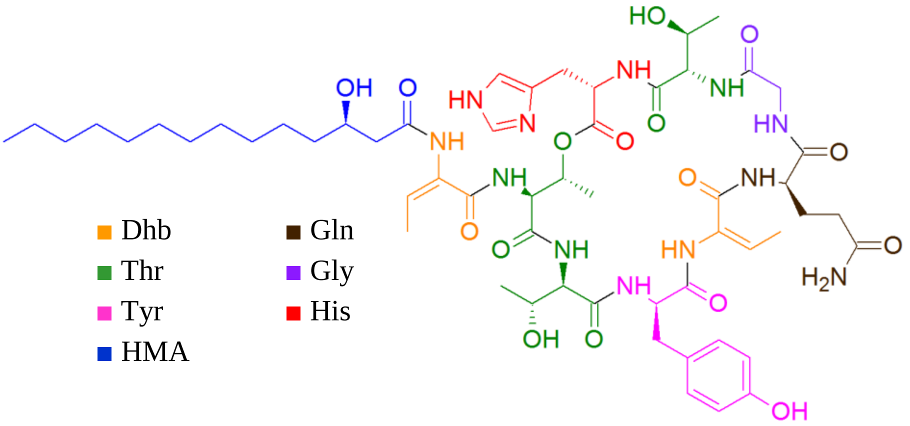

Figure S1 Structure of jagaricin (HMA = β-hydroxymyristic acid, Dhb = dehydrobutyrine).

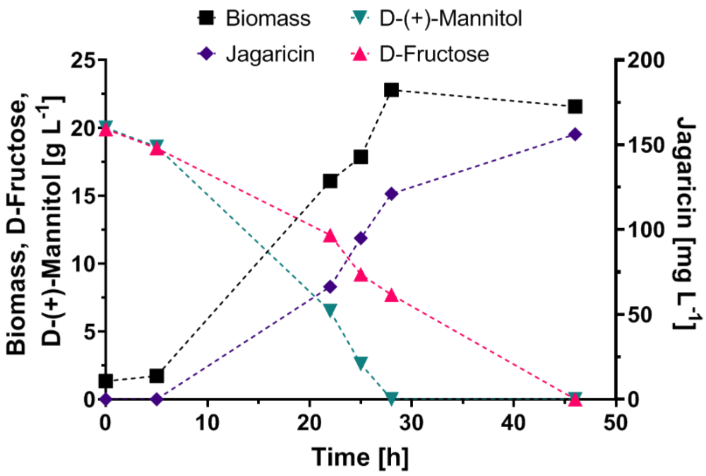

Figure S2 Cultivation of *Janthinobacterium agaricidamnosum* DSM 9628 for jagarcin production.

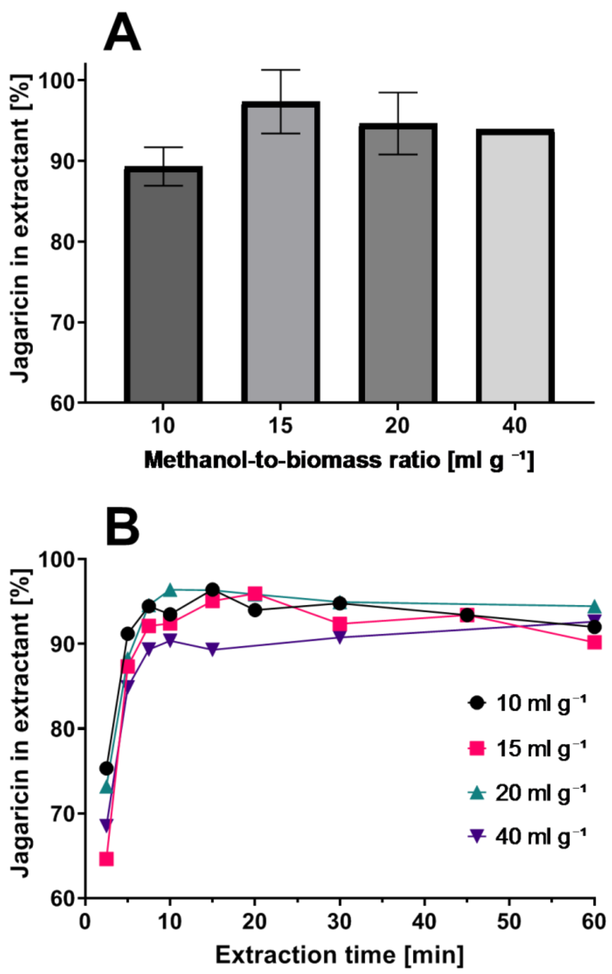

Figure S3 Extraction process of jagaricin using methanol. **(A)** Variation of methanol-to-biomass-ratio; **(B)** Extraction kinetic of jagaricin for different methanol-to-biomass-ratios.

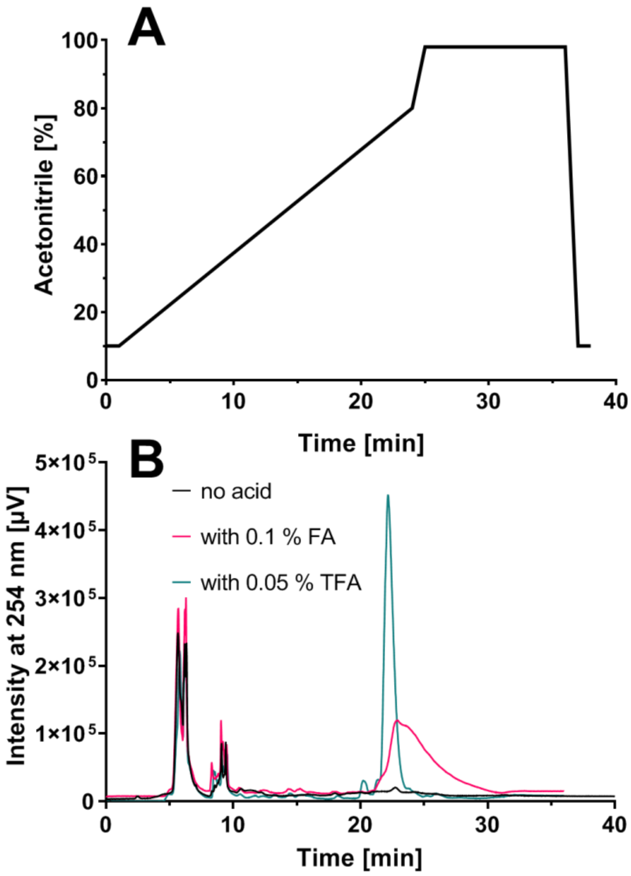

Figure S4 Preparative HPLC: **(A)** Gradient of water-acetonitrile with a flow rate of 20 ml min^{-1}; **(B)** Chromatograms of different runs with 0.1 % formic acid (FA) or 0.05 % trifluoroacetic acid (TFA) and without acid addition for reference.

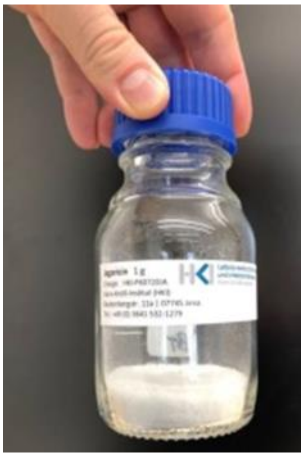

Figure S5 Final product with a purity of 96.9 %.
